# Selective Attention Measurement of Experienced Simultaneous Interpreters Using EEG Phase-Locked Response

**DOI:** 10.3389/fnhum.2021.581525

**Published:** 2021-06-07

**Authors:** Haruko Yagura, Hiroki Tanaka, Taiki Kinoshita, Hiroki Watanabe, Shunnosuke Motomura, Katsuhito Sudoh, Satoshi Nakamura

**Affiliations:** Division of Information Science, Nara Institute of Science and Technology, Nara, Japan

**Keywords:** simultaneous translation, EEG, inter-trial coherence, interpreter's advantage, selective attention, auditory steady-state response

## Abstract

We quantified the electroencephalogram signals associated with the selective attention processing of experienced simultaneous interpreters and calculated the phase-locked responses evoked by a 40-Hz auditory steady-state response (40-Hz ASSR) and the values of robust inter-trial coherence (ITC) for environmental changes. Since we assumed that an interpreter's attention ability improves with an increase in the number of years of experience of simultaneous interpretation, we divided the participants into two groups based on their simultaneous interpretation experience: experts with more than 15 years of experience (E group; *n* = 7) and beginners with <1 year (B group; *n* = 15). We also compared two conditions: simultaneous interpretation (SI) and shadowing (SH). We found a significant interaction in the ITC between years of SI experience (E and B groups) and tasks (SI and SH). This result demonstrates that the number of years of SI experience influences selective attention during interpretation.

## 1. Introduction

Simultaneous interpretation (SI), which is also called extreme multitasking (Cowan, 1999; Camayd-Freixas, 2011), requires interpretation at the same speed as a speaker's utterances. In SI, the next utterance begins before the current interpretation is finished. SI and its cognitive mechanism focus on how simultaneous interpreters overcome their cognitive load (Gile, 1999; Cowan, 2000; Mizuno, 2005). Recent reports argue that as a person gains SI experience, his/her overall cognitive function also improves, which changes the brain functions and structures (Rinne et al., 2000; Elmer et al., 2011; Hervais-Adelman et al., 2015; Elmer and Kühnis, 2016; Van de Putte et al., 2018). Therefore, brain mechanisms are attracting research attention because they offer new possibilities for training methods that might improve human cognitive functions. In this study, we quantified the differences in brain activity patterns between experienced and beginner interpreters using electroencephalograms (EEGs) in realistic environments during SI. We set two experimental conditions: SI and simultaneous shadowing (SH). In SH, the participants simultaneously repeat from Japanese to Japanese.

Research interest describes interpreters' cognitive processing (Gile, 1999; Cowan, 2000; Mizuno, 2005). A well-publicized psychological model during SI is explained by selective attention processing during multitasking (Cowan, 1999, 2000). Cowan's working memory model, which focuses on the processing of attention switching, is an especially common psychological model for describing cognitive load during simultaneous interpretation (Cowan, 1999, 2000). According to it, simultaneous interpreters are constantly switching attention from one aspect to another, focusing on each task (listening, interpreting, speaking, etc.), not exactly simultaneously, but conterminously a little later. However, the amount of information is limited on which humans can focus at one time. And when this amount exceeds a limit, the cognitive load increases rapidly. In this way, Cowen et al. proposed a flexible model that fits the reality of cognitive load and attention switching that change from moment to moment according to the speed of the speaker's speech during SI. In addition, switching attention in their model refers to selective attention and the relationship between attention and cognitive load (Cowan, 1999, 2000; Mizuno, 2005).

Many such studies focus on the “bilingual advantage,” which is the ability to switch attention between native and non-native languages (Ardila, 2003; Abutalebi et al., 2012; Woumans et al., 2015; Zhang, 2018), and the “interpreter's advantage,” which requires more frequent language switching than bilingual approaches (Dong and Xie, 2014; Morales et al., 2015; Strobach et al., 2015; Dong and Liu, 2016; Babcock et al., 2017). Many studies have clarified the brain's SI mechanism. The brain function models of experienced simultaneous interpreters have been investigated using functional magnetic resonance imaging (fMRI) and positron emission tomography (PET) with excellent spatial resolution (Rinne et al., 2000; Elmer et al., 2011; Hervais-Adelman et al., 2015; Elmer and Kühnis, 2016; Van de Putte et al., 2018). When research on SI brain imaging was first reported, the left prefrontal cortex was the focus of an experiment that compared the interpretation direction (native to target language, target to native language, or shadowing in native language; Rinne et al., 2000; Elmer and Kühnis, 2016). Hervais-Adelman proposed an adaptive control hypothesis by improving an adapted control network for bilingual speakers (Hervais-Adelman et al., 2015). According to their work, in well-trained SI, brain networks maintain quick attention switching between two languages that need to be preserved. As suggested by previous work, attention switching in experienced SI plays an important role in the parietal cortex and the dorsal striatum, which is related to adapted control networks (Rinne et al., 2000; Elmer et al., 2011; Hervais-Adelman et al., 2015; Elmer and Kühnis, 2016; Van de Putte et al., 2018). The cerebellum is involved as a function that automates complex speech behavior during SI (Rinne et al., 2000; Ackermann, 2008; Hervais-Adelman et al., 2015). Most of these studies used fMRI and PET. However, body movements and measurement sounds cause much noise. In recent years, the development of active electrodes and portable EEG has made it possible to measure brain function in situations closer to realistic environments, such as while walking (Yokota and Naruse, 2015; Yokota et al., 2017).

A remarkable EEG experiment by Koshkin et al. (2018) during SI extracted N1 (the negative-amplitude response about 100 ms after the stimuli onset) and P1 (the positive-amplitude response after about 50–100 ms), which are early event-related potential (ERP) components related to selective attention. In this experiment, the probe sounds (tone pips) were embedded in the sound stimuli at regular intervals to extract the early ERP components evoked from probe sounds. The responses of the early ERP components changed as the attention resource decreased since the SI difficulty increased. On the other hand, measuring EEG and fMRI is often complicated during SI due to such environmental noise on signals as mouth and hand movements, blinking, and interpreter's voices. In addition, most previous EEG and fMRI experiments on the brain activity during SI used averaging and a block design to compare the stimuli presented repeatedly at short intervals with seconds or milliseconds. Therefore, it is difficult to completely detect the attention switching process in SI that occurred during continuous interpretation (Gile, 1999; Cowan, 2000; Mizuno, 2005).

We used the 40-Hz auditory steady-state response (40-Hz ASSR), which is robust against various environmental changes and provides realistic environments during SI (Tiitinen et al., 1993; Ackermann et al., 1998). The 40-Hz ASSR is the brain responses evoked by repeated auditory stimuli, whose source location is reported to be in the brain stem, in the thalamus, and in the auditory cortex (Herdman et al., 2002; Farahani et al., 2019, 2021). Forty Hertz ASSR is a highly reproducible signal whose test–retest reliability was validated by EEG and MEG as a biological indicator for schizophrenia and other disorders (McFadden et al., 2014; Legget et al., 2017; Hirano et al., 2020). In addition, since 40-Hz ASSR is a sequence of pulse signals that can be simultaneously presented as the target stimulus (voice stimulus, visual stimulus, etc.), only the 40-Hz pulse signal is synchronized with the EEG measurements. Therefore, the target stimulus does not need to be edited into short segments and can be presented in its original form. The 40-Hz ASSR can be simply quantified by a phase-locked index called inter-trial coherence (ITC). With 40-Hz ASSR and ITC, Yokota et al. quantified the cognitive function of an n-back task that was performed while walking to simulate a realistic environment (Yokota and Naruse, 2015; Yokota et al., 2017). To detect the bilingual advantage associated with the ability to overcome cognitive load, the audio source must be presented continuously, as in a realistic environment during SI. They used the ITCs from the 40-Hz ASSR to assess whether a new portable EEG system with active electrodes can be used in such realistic environments as walking. On the other hand, SI tasks have a high cognitive load, which is characterized by continuously presented target sounds that must be translated. Therefore, the 40-Hz ASSR, which measures EEG signals by focusing on the 40-Hz ASSR signal presented at the same time without editing the audio, is a very effective index for verifying ability during SI.

On the other hand, gamma-band oscillation has brain activity at various sites related to several types of cognitive processing, including memory, emotion, and attention (Jensen et al., 2007; Herrmann et al., 2010; Başar, 2013). The gamma-band rhythm results from the coordinated interaction of excitement and the inhibition of cerebral cortical activity (Buzśaki and Wang, 2012; Neske and Connors, 2016). The dysfunction of this gamma-band coordinated interaction is associated with the cognitive dysfunction of several diseases, such as schizophrenia (Buzśaki and Wang, 2012; Neske and Connors, 2016). The gamma band oscillation is also associated with cognitive load, which increases as difficulty rises (Howard et al., 2003; Basar-Eroglu et al., 2007; Van Vugt et al., 2010). By comparing groups of schizophrenia patients and healthy subjects who performed tasks that have different difficulty levels, only the gamma-band oscillations increases in the healthy group as the difficulty of the cognitive load intensifies (Basar-Eroglu et al., 2007). In addition, the 40-Hz ASSR is modulated by selective attention processing (Tiitinen et al., 1993; Müller et al., 2009; Mahajan et al., 2014; Manting et al., 2020). Schizophrenia patients reportedly have a reduced 40-Hz ASSR compared to healthy subjects (Thuné et al., 2016; Hirano et al., 2020). Measuring 40-Hz ASSR while performing several tasks with different cognitive loads decreases the response at higher cognitive loads (Griskova-Bulanova et al., 2011; Yokota and Naruse, 2015; Yokota et al., 2017). More recently, a study argued that 40-Hz ASSR, selective attention processing, and such associated sites as frontal-temporal-parietal prompt wider cortical responses (Manting et al., 2020).

Since SI is multitasking, it must inhibit unnecessary stimuli input so that attention to the target language works effectively. Previous studies reported that the selective attention processing of participants with SI experience improved more than participants without or less SI experience (Strobach et al., 2015; Henrard and Daele, 2017; Van der Linden et al., 2018). In other words, if a person is interpreting while listening to a 40-Hz pulse signal, we assume that more attention is paid to SI, and the attention to the signal is inhibited. This reduces the 40-Hz ASSR. Yokota et al. also reported that ITC decreases as the difficulty of the n-back task increases and the attention to the 40-Hz pulse signal decreases (Yokota and Naruse, 2015; Yokota et al., 2017). Therefore, we hypothesize that ITC decreases as SI experience increases. Accordingly, we expect a significant interaction between the selective attention to SI and the amount of SI experience. The SI condition is multitasking with a higher cognitive load than the SH condition, and simultaneous interpreters need to pay more selective attention to the target language. Since SH requires less attention processing than SI, the SH 40-Hz ASSR will be lower than SI.

Therefore, the following two points comprise the purpose of this study:

1. Verifying how selective attention processing modulated by 40-Hz ASSR is affected by the years of simultaneous interpreting experience;
2. Quantifying selective attention processing modulated by 40-Hz auditory steady-state responses during simultaneous translation (SI) in a realistic environment.

This paper is an extension of our previous conference proceeding (Yagura et al., 2020). In it, we compared the ITCs from 40-Hz ASSR phase-lock responses between groups with different years of experience. We analyzed the frontal electrodes over the frontal lobe, which is involved in the SI attention switching process (Rinne et al., 2000; Elmer et al., 2010; Hervais-Adelman and Babcock, 2019). In addition, to confirm the 40-Hz ASSR properties in these datasets, we analyzed three regions of interest (ROI) by frontal, temporal, and parietal electrodes.

## 2. Methods

### 2.1. Participants

We separated 22 professional, Japanese female interpreters into groups of Experienced (experts: E group; seven participants with over 15 years of SI experience) and Beginners (beginners: B group; 15 participants with at least one year of SI experience). From statistical Welch's *t*-test results, we found no differences in the ages of the two groups [age; *t*_(21)_ = 1.55, *p* = 0.161, E group; *n* = 7, mean = 56.71, sd = 8.88, B group; *n* = 15, mean = 51.2, sd = 4.33]. None had hearing-related problems or any history of psychiatric problems (Hirano et al., 2015, 2020; Thuné et al., 2016). All the participants were registered with a translation agency, which determined the E or B group based on its own criteria. We did not scientifically determine their differences in years of experience. Instead, we subjectively evaluated the SI difficulty and objectively evaluated the differences based on the years of experience of the interpreters.

### 2.2. Sound Stimuli

**Figure 1:**
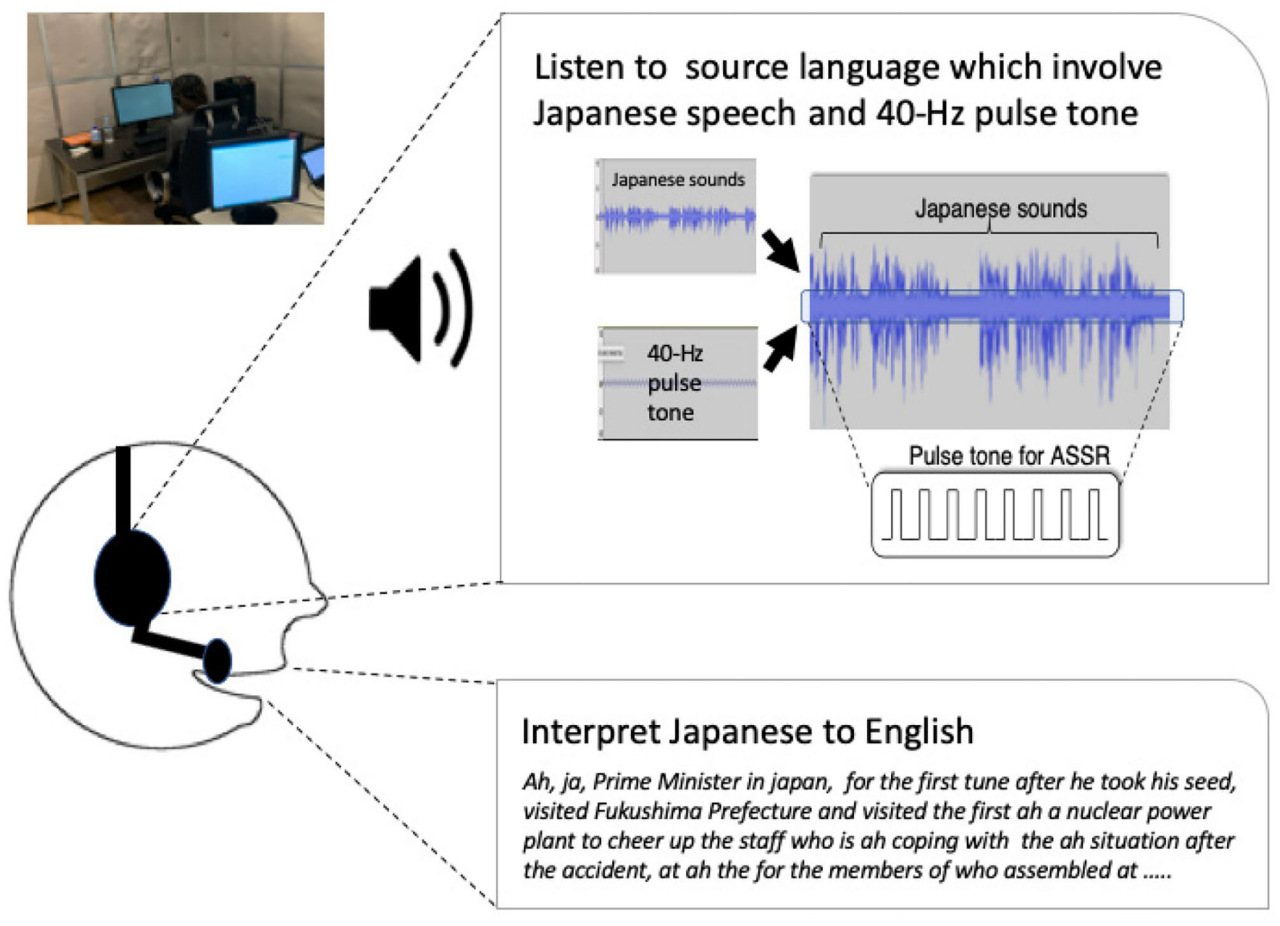
Forty Hertz pulse tones to elicit 40-Hz auditory steady-state responses (40-Hz ASSR) and Japanese radio news sounds were synthesized in stereo and presented to their ears.

We prepared eight topics from the natural speech data of NHK radio news because it is Japan's most representative news broadcast (Shimizu et al., 2014; Table 1). We used a 40-Hz rate pulse tone to elicit the 40-Hz ASSRs and the speech sounds with the radio news based on previous studies (Yokota and Naruse, 2015; Figure 1). The auditory repetitive stimuli were 40-Hz clicks (sampling rate: 8,192 Hz), which consisted of a 10-ms pulse width that was repeated every 25 ms. The auditory stimulus was presented in 15-s trial intervals and durations of 60 s. The number of trials was randomly presented 16 times: 8 times for 8 topics under the simultaneous interpretation condition and 8 times for 8 topics under the shadowing condition. The sound pressure was normalized with maximum amplitude. The pulse tone's pressure level was edited by a speech therapist to evaluate whether it could be heard adequately and whether it felt uncomfortable at 5% of the maximum amplitude of the news sounds. The 40-Hz pulse tone and the news sounds were synthesized in stereo and presented to the subject's ears by insert earphones (ER1) at a sound pressure of 70–85 dB. We created audio files with audio-editing software called Audacity.

**Table 1:** Example of part of Japanese radio news.

	**Contents of sound stimuli translated into English from Japanese**
Topic 2	Japanese Prime Minister Abe visited Fukushima Prefecture for the first time and encouraged the workers to resolve the disaster at the Tokyo Electric Power Company Fukushima Daiichi Nuclear Power Station.
Topic 6	The Chuo Expressway has been closed due to an accident in an up route of the Sasako tunnel. Unfortunately, an up route and a down route near the site are closed, but the down route is scheduled to reopen today at about 2:00 p.m. and face-to-face traffic will continue.

### 2.3. Task Sequences

**Figure 2:**
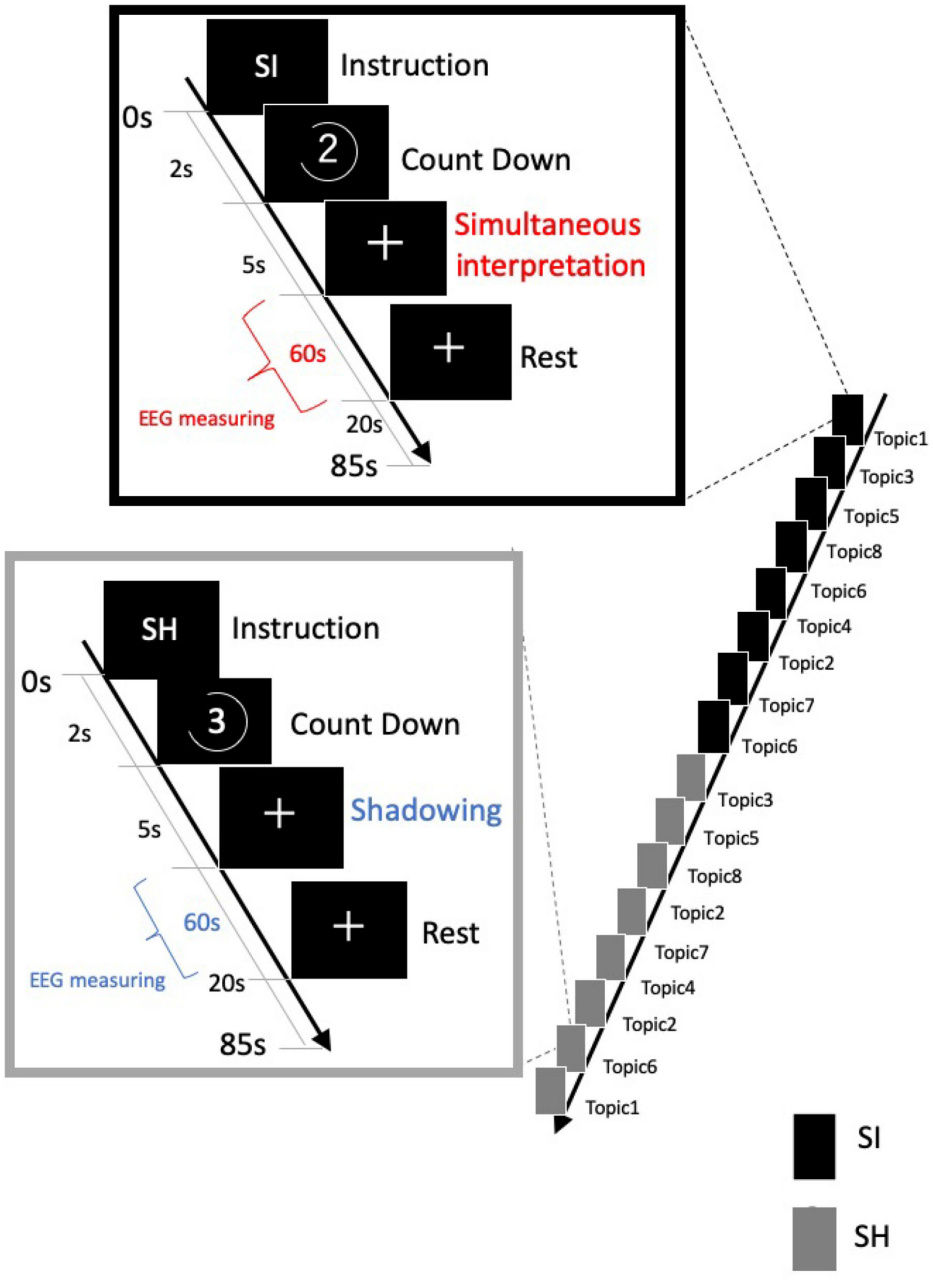
Our task consisted of two conditions: simultaneous translation from Japanese to English [simultaneous interpretation (SI) condition] and shadowing that immediately repeats Japanese [simultaneous shadowing (SH) condition]. Under both conditions, eight 60-s news topic sounds were randomly presented. Identical voice stimuli were used under both conditions.

Our task consisted of two conditions: simultaneous translation from Japanese to English (SI condition) and shadowing that immediately repeats the Japanese (SH condition: without SI; Figure 2). We randomly presented news on eight topics under both conditions. The difference between the SI and SH conditions is for investigating whether interpreters pay attention to SI. The stimulus was created using presentation software from Neurobehavioral Systems (Version 18.0, Neurobehavioral Systems, Inc., Berkeley, CA, www.neurobs.com).

### 2.4. Subjective Evaluations

After the EEG experiment, the subjects completed subjective evaluation sheets for five questions on each topic: the content of eight bits of Japanese source audio. The following are the contents of each question:

Question 1: How good was your interpretation? (1: Very good, 5: not very good)
Question 2: How familiar are you with this topic? (1: Very familiar, 5: unfamiliar)
Question 3: How was the voice speed? (1: slow, 5: fast)
Question 4: Was it easy to listen to the audio? (1: easy, 5: difficult)
Question 5: How would you rate the overall difficulty? (1: easy, 5: difficult)

### 2.5. EEG Data Acquisition and Prepossessing

We recorded the EEG data acquisition and prepossessing of the EEG signals with a Cognionics Quick-30 Dry EEG headset with 29 electrodes (excluding one for the reference electrode; Camayd-Freixas, 2011). We used an EEGLAB automatic processing pipeline, and the recorded signals were FIR-bandpass filtered from 1 to 50 Hz at a sampling rate of 500 Hz. The EEG signals were referenced by subtracting the average signals of A1 and A2. The ground electrode was placed in the center between Fp1 and Fp2. We performed an independent component analysis (ICA) to eliminate eye movements and eye blinks related to the saccadic spike artifact using EEGLAB (Keren et al., 2010; Carl et al., 2012). No other artifacts were eliminated. All the EEG signals were prepossessed using MATLAB (Math Works, Natick, MA, USA).

### 2.6. ITC

**Figure 3:**
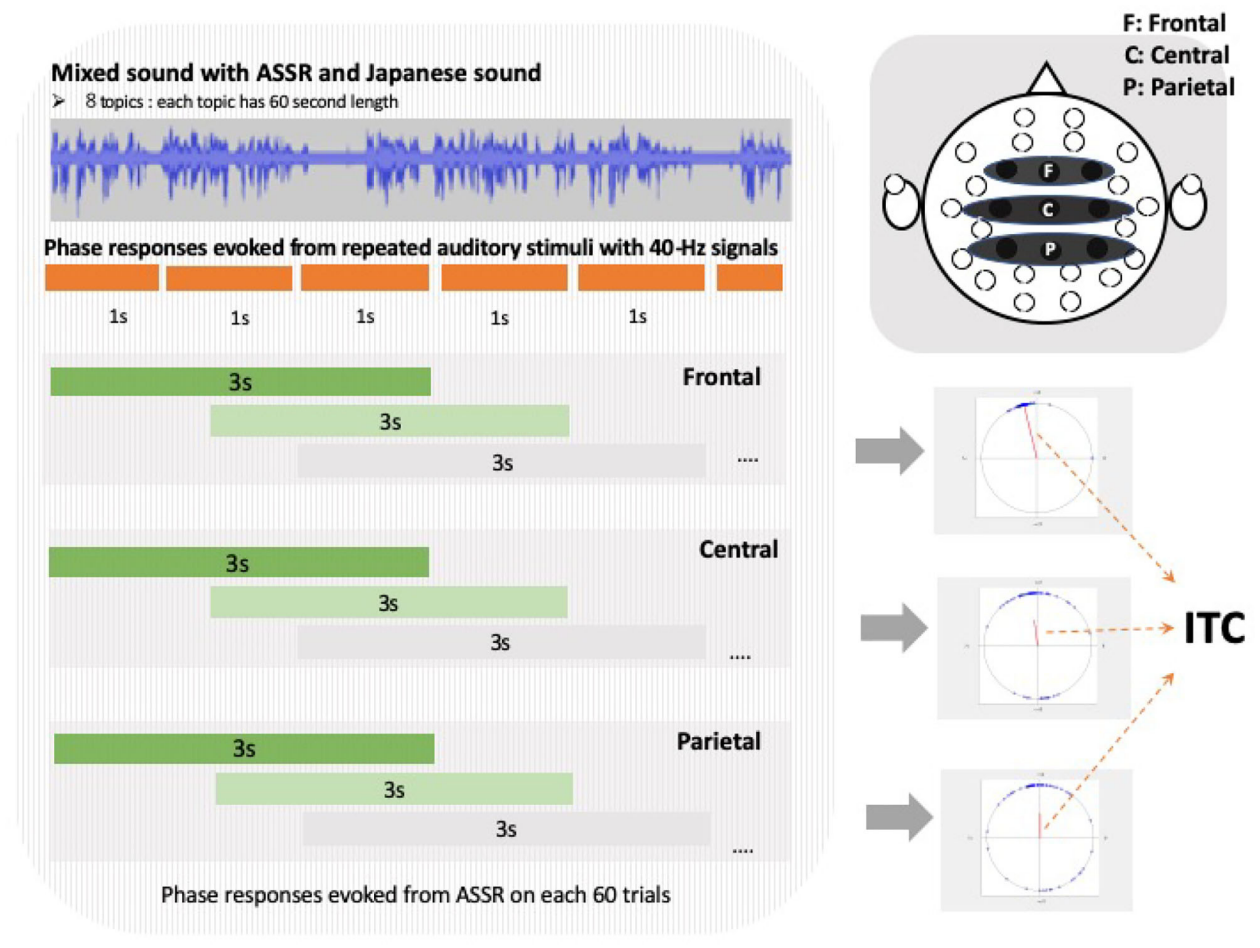
Left figure shows inter-trial coherence (ITC) calculation method of each regions of interest (ROI). Each red line in center of three circles in lower right diagram is direction and magnitude of average composite vector as ITC, and blue dots at edges of circle indicate phases in each trial.

To quantify the selective attention processing using EEG signals related to the amount of SI experience, we extracted the phases at 40-Hz and calculated the ITC based on previous research (Griskova-Bulanova et al., 2011; Yokota et al., 2017). Although wavelet transform detects the time and frequency changes in detail, in our experiments the time frequency was fixed at a constant 40 Hz, and so we calculated the ITCs by averaging the phases of each trial using a short-time Fourier transform (Tallon-baudry et al., 1996; Roach and Mathalon, 2008). We divided the continuous EEG data into 3-s trials and shifted them by 1 s (Figure 3). We performed a Fourier transform at 40 Hz for each electrode and calculated the ITC based on the following equation:

$$ITC\left[ch\right]=|\frac{\sum_{k=1}^{K}exp\left(j\theta_{k}^{f}\left[ch\right]\right)}{K}|,$$

where *f* is a frequency, *el* is the electrode number, $\theta_{k}^{f}$ is the phases of frequency f and electrode el, *k* is a trial number, and *K* is the number of trials. The 40-Hz ASSR responses originate from the primary auditory cortex. ASSR's attention modulation has also been observed in the frontal, temporal, and parietal regions (Herdman et al., 2002; Farahani et al., 2019; Manting et al., 2020). In our work, based on these previous studies (Griskova-Bulanova et al., 2011), we analyzed ITC using ROIs that were selected for the frontal, temporal, and parietal electrodes to include or border the frontal or primary auditory cortex (Griskova-Bulanova et al., 2011). The ITCs were calculated for each news topic over the three regions of interest (ROIs): frontal (F3, Fz, and F4), central (C3, Cz, and C4), and parietal (P3, Pz, and P4) (Griskova-Bulanova et al., 2011; Yokota et al., 2017). The ITCs were averaged over the electrodes in each ROI (Yokota et al., 2017; Figure 3).

### 2.7. Statistical Analysis

We performed a three-factor mixed ANOVA on YEAR, TASK, and ROI to investigate the interaction of ITC between YEAR (E and B groups) and TASK (SI and SH) on each ROI: frontal, central, and parietal.

For the subjective evaluations, to determine the difficulty of our two tasks under each condition based on experience for subjective evaluations, we performed a Wilcoxon rank-sum test with the E and B groups. Since the contents of the five questions on the subjective evaluation values are unrelated, they are considered independent variables for each question.

## 3. Results

### 3.1. Subjective Evaluation

**Figure 4:**
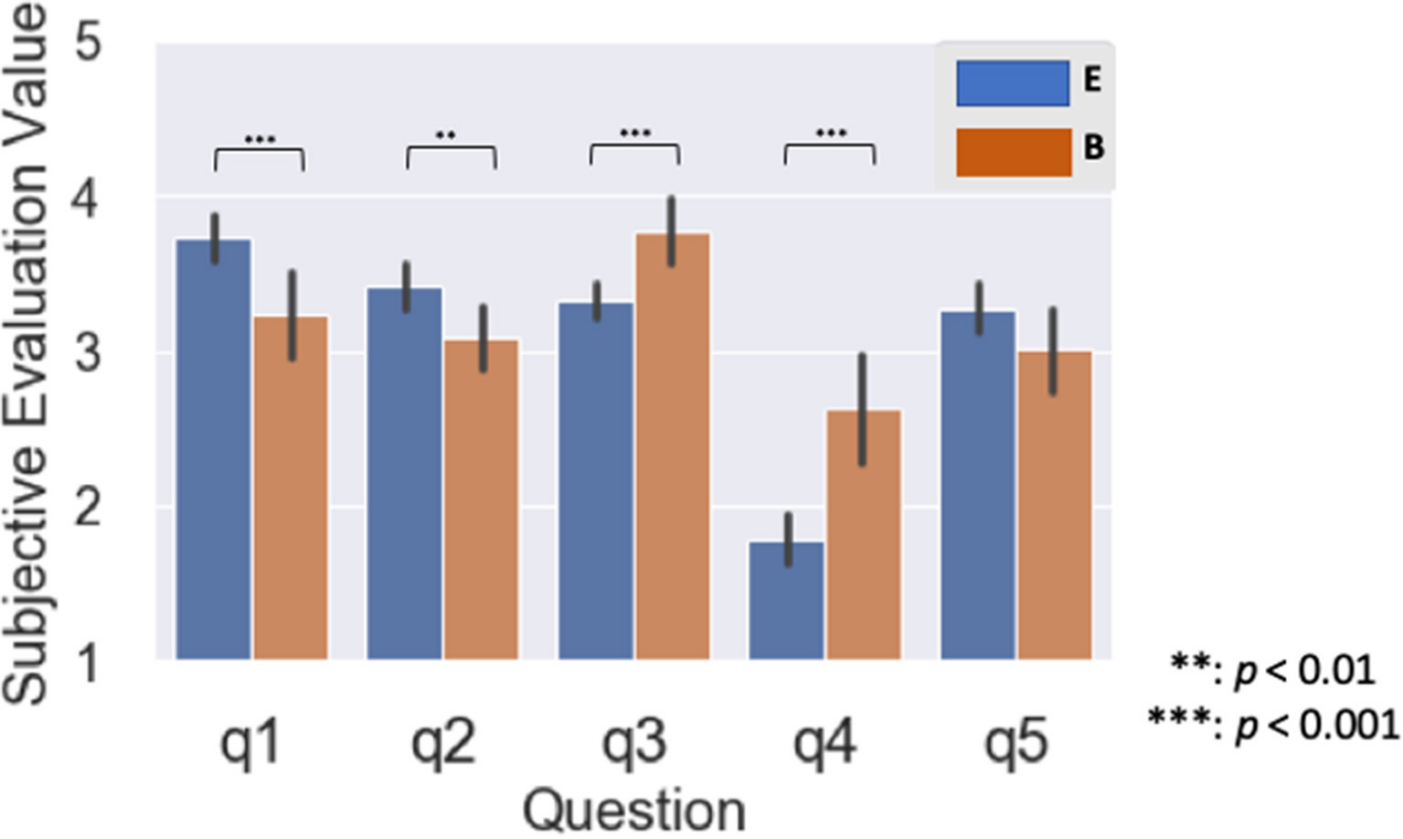
Mean of subjective evaluation value for two groups with different years of simultaneous interpretation (SI) experience: Experienced group with over 15 years was represented as E, and a beginner group with <1 year was represented as B. All questions were rated on five levels of interpretation difficulty: level 1: participants felt interpretation was easy; level 5: participants felt interpretation was difficult.

We performed a Wilcoxon rank-sum test with the two SI experience groups for all five questions (Figure 4). For Questions 1 and 2, the subjective evaluation value for the B group significantly exceeded the E group (q1: *p* < 0.001, q2: *p* = 0.021; Figure 4). For Questions 3 and 4, the subjective evaluation value for the E group significantly exceeded the B group (q3: *p* < 0.001, q4: *p* < 0.001; Figure 4). On the other hand, we identified no significant differences of subjective evaluation values between the groups for Question 5 (q5: *p* = 0.23; Figure 4).

### 3.2. ITC in Each ROI

**Figure 5:**
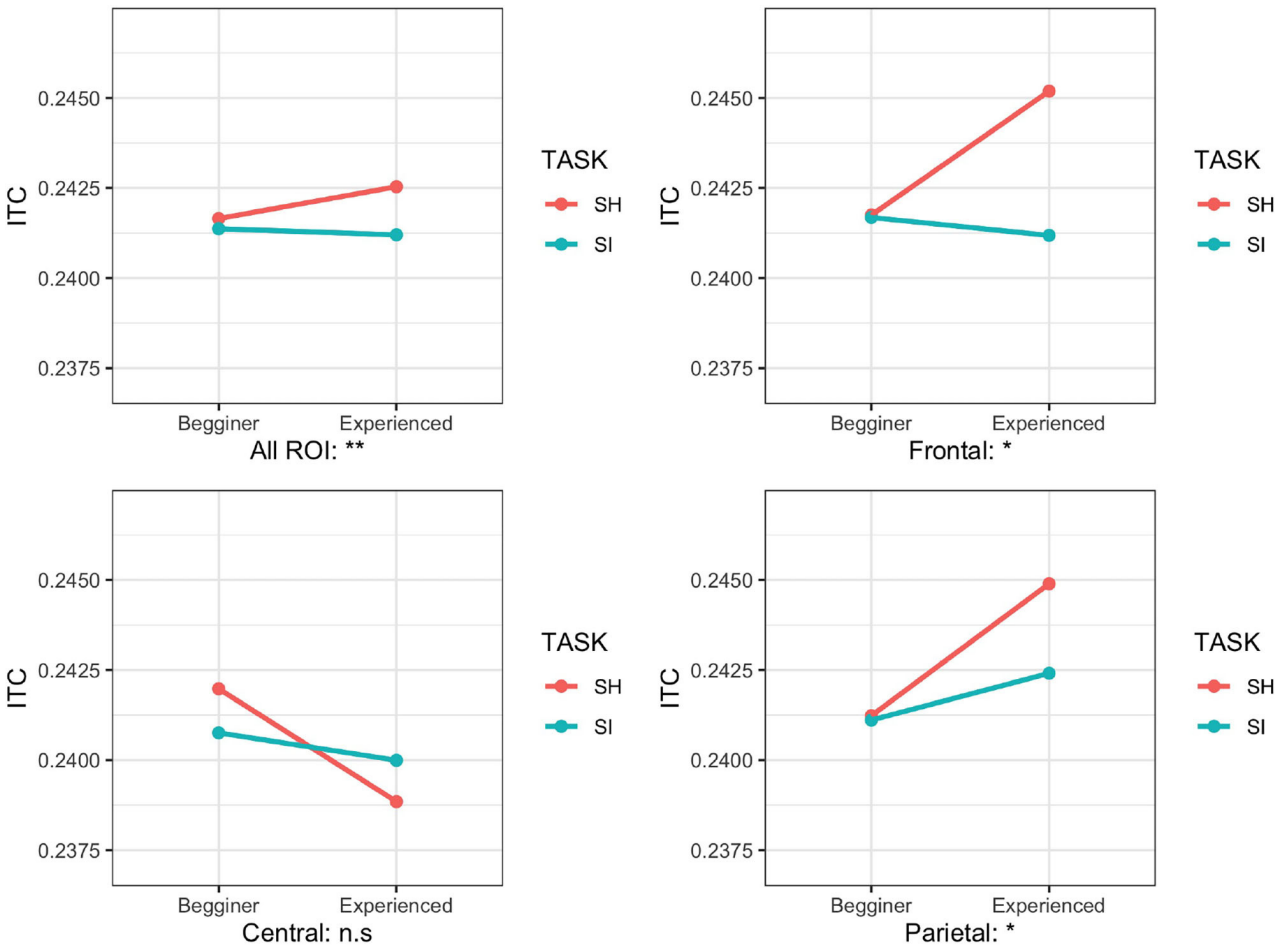
Mean inter-trial coherence (ITC) values for different regions (all regions of interest [ROIs], frontal, central, and parietal) in two task conditions [simultaneous interpretation (SI) and simultaneous shadowing (SH)] and two groups (beginner group/B and experienced group/E). **p < 0.01, *p < 0.05.

We performed a three-factor mixed ANOVA for the ITC to investigate the interaction between YEAR (E and B groups) and TASK (SI and SH). Since the normality of the ITCs was rejected in each ROI (*p* < 0.001), we performed an adjusted rank transform test (ART) with the non-parametric method to analyze the interaction (Leys and Schumann, 2010). We applied ART to the ITC with YEAR (E and B groups), TASK (SI and SH), and ROI (F: frontal, C: central, and P: parietal). ART identified the main effects of the TASK [*F*_(1,1044)_ = 6.76; *p* = 0.0004, partial $\eta_{p}^{2}$ = 0.02] and ROI [*F*_(2,1044)_ = 9.05; *p* = 0.001, partial $\eta_{p}^{2}$ = 0.004]. A significant interaction was shown in YEAR and TASK [*F*_(1,1044)_ = 10.70; *p* = 0.001, partial $\eta_{p}^{2}$ = 0.007] and YEAR and ROI [*F*_(2,1044)_ = 3.29; *p* = 0.03, partial $\eta_{p}^{2}$ = 0.008; Figure 5].

To investigate which location showed a clear interaction by YEAR (E and B groups) and TASK (SI and SH), we divided the data into three ROI parts (frontal, central, and parietal) and performed ART with a 2-way mixed ANOVA on YEAR (E and B groups) and TASK (SI and SH). The main effects of TASK were significant on the central and the parietal electrodes [central: *F*_(1,328)_= 4.48; *p* = 0.03, partial $\eta_{p}^{2}$ = 0.02, parietal: *F*_(1,328)_= 12.94; *p* = 0.0001, partial $\eta_{p}^{2}$ = 0.04]. ART revealed a significant interaction between YEAR and TASK on all ROIs, the frontal and parietal electrodes [all ROIs, *F*_(1,1044)_ = 9.05; *p* = 0.002, partial $\eta_{p}^{2}$ = 0.007; frontal, *F*_(1,328)_ = 5.33; *p* = 0.01, partial $\eta_{p}^{2}$ = 0.01, *F*_(1,328)_ = 4.78; *p* = 0.029, partial $\eta_{p}^{2}$ = 0.008; Figure 5]. However, on the central electrodes, the interaction between YEAR and TASK was not significant [*F*_(1,328)_ = 1.5; *p* = 0.22, partial $\eta_{p}^{2}$ = 0.001].

To determine which TASKs showed a clear difference in each electrode where the interaction showed significance, we tested Wilcoxon's rank-sum test by Bonferroni. It revealed that the ITCs of the E group under the SH conditions increased significantly more than the SI condition (all ROIs: W = 10,194, *p* < 0.04, frontal: W = 1,229, *p* = 0.04, parietal: W = 980, *p* = 0.0006); no significant difference of the ITCs between the SH and SI conditions on the B group was shown on the frontal and the parietal electrodes (all ROIs: W = 10,194, *p* < 0.04, frontal: W = 61,547, *p* = 0.2437, parietal: W = 6,195, *p* = 0.06).

## 4. Discussion

The results of the subjective evaluation values reflect the amount of SI experience. The following questions were related to SI difficulty: q1 (satisfaction with interpretation), q2 (topic familiarity), and q5 (overall difficulty of simultaneous interpretation). In q1 and q2, the subjective ratings were significantly higher in the B group than in the E group, and the B group with less experience had more difficulty while interpreting (Figure 4). In q2, the difference in experience may have been reflected in the interpreter's specialty, which may also be related to word familiarity; when it is high, recalling the meaning of words is easier, which obviously reduces the difficulty of interpreting (Gardner et al., 1987; Coane and Balota, 2010; Koshkin et al., 2018). Perhaps the difficulty felt by the B group interpreters was related to the topic field. In q5, we found no significant difference between the E and B groups, probably because the overall difficulty level of the question content included different views on the question. For example, for q3 (ability to comprehend the original speech speed) concerning the speed of the voice, the E group perceived it to be faster than the B group. The E group knew that the speed of the news voice was faster than the other topics, such as conferences and presentations. But the B group participants with less than a year's SI experience didn't know that the speed of a news broadcast is faster than the other topics; they thought that the voice's speed itself was either normal or slow. In q4 (ease of listening), the E group thought the listening was more difficult than did the B group in terms of audibility. Most E group interpreters wanted to use their own headphones. The inexperienced B group just thought that the sound pressure during the EEG experiment was easy to hear. Before the experiment, we adjusted the sound pressure for them. In this way, we confirmed that the subject evaluation values for SI represent the differences due to SI experience.

In our experiments, the interaction of the ITCs between year and task was observed in the frontal and parietal regions (Figure 5). The ITC for the E group under the SH condition increased more than in the SI condition. On the other hand, the B group showed almost no significant differences between tasks. Previous studies reported that ITCs decrease with increasing difficulty during tasks of different difficulty while listening to 40-Hz pulses (Griskova-Bulanova et al., 2011; Yokota and Naruse, 2015; Yokota et al., 2017). As SI experience grows, interpreters focus on the target stimuli during multitasking (Strobach et al., 2015; Henrard and Daele, 2017; Van der Linden et al., 2018). Under the SI condition, we assume that more attention is paid to SI, and the attention to the 40-Hz pulse signal is inhibited. On the other hand, the SH task did not include any simultaneous interpretation, which is advanced multitasking. Therefore, we assume that the inhibition of attention to the 40-Hz pulse was smaller than SI, and the ITC increased more than SI. Previous studies compared the gamma-frequency oscillation during n-back tasks between healthy subjects and patients with schizophrenia, and in the schizophrenia group, there is no difference in gamma-frequency oscillation between simple and difficult tasks. In healthy participants, a significant increase in gamma-frequency oscillation was observed between tasks (Basar-Eroglu et al., 2007; Jensen et al., 2007). This implies that schizophrenia sufferers struggle to adjust their attention based on a task's difficulty. For the B group in our experiment, the increased task load may prevent simultaneous interpreters from properly controlling their attention distribution. This experiment suggests that task difficulty and years of experience impact the attention modulation of 40-Hz ASSR.

The interaction of ITCs between YEAR and TASK was observed in the frontal and parietal regions (Figure 5). The location of the source of the 40-Hz ASSRs was found in the brainstem and auditory cortex (Herdman et al., 2002; Farahani et al., 2019). In addition, the attention modulation of 40-Hz ASSRs is involved in the temporal and parietal regions (Manting et al., 2020). This modulation extended from the frontal region to the parietal and temporal regions. Experiments examining the brain activity of many simultaneous interpreters, such as fMRI, have concluded that the parietal and frontal regions are associated with SI training (Rinne et al., 2000; Hervais-Adelman et al., 2015; Elmer and Kühnis, 2016; Klein et al., 2018). Determining the direct effect of SI training on the parietal and frontal regions requires further validation on EEG signals.

In this study, we quantified the EEG signals associated with the selective attention processing of experienced simultaneous interpreters using ITC in a realistic environment during SI. We identified a significant interaction in the ITC between years of SI experience (E and B groups) and tasks (SI and SH). This result suggests that years of SI experience influence selective attention during interpretation.

## 5. Conclusions

For experienced SI, we quantified with EEG their selective attention, which includes superior attention switching ability. We also conducted EEG measurements under conditions close to the realistic environments of interpreters using the ITCs of 40-Hz phase synchronization signals, which are strong against environmental changes. The ITCs of experienced SI users during the SI condition significantly increased more than during the SH on the E group. Based on these results, we conclude that the 40-Hz ASSR is a suitable indicator during SI in realistic environments. However, in this experiment, since we only compared one SI direction from Japanese (native language) to English, further verification is needed using multiple language pairs. Another limitation of this study is the inequality in the number of subjects between the experienced SI (*n* = 7) and beginners (*n* = 15) because experienced SIs are much more scarce. Since the gamma band interacts with other frequency oscillations (e.g., delta band, theta band, and beta band), our future work will investigate the relationship with oscillations other than the gamma band. Further examination of the effect caused by linguistic and semantic structure is also necessary, including linguistic analysis. The 40-Hz ASSR will also be used to assess attention-deficit hyperactivity disorder and schizophrenia. Future work will experimentally expand our range of applications.

## Data Availability Statement

The raw data supporting the conclusions of this article will be made available by the authors, without undue reservation.

## Ethics Statement

The studies involving human participants were reviewed and approved by The Research Ethics Committee of NAIST. The patients/participants provided their written informed consent to participate in this study. Written informed consent was obtained from the individual(s) for the publication of any potentially identifiable images or data included in this article.

## Author Contributions

HY, HT, TK, HW, and SM performed the experiments and the data analysis and conceived the methodology and the phase-locked responses that were extracted by 40-Hz ASSR. HY, HT, HW, and TK performed the EEG data collecting. HY, HT, and HW conceived the entire experiment design. HY, HT, and SN analyzed and discussed the results. KS and SN provided information about the field of interpreting. HY wrote the manuscript. SN is the principal investigator of the project and directs the whole research. All authors contributed to the article and approved the submitted version.

## Conflict of Interest

The authors declare that the research was conducted in the absence of any commercial or financial relationships that could be construed as a potential conflict of interest.
